# Protein Phosphatase 2A Mediates Oxidative Stress Induced Apoptosis in Osteoblasts

**DOI:** 10.1155/2015/804260

**Published:** 2015-10-11

**Authors:** Chong-xin Huang, Bo Lv, Yue Wang

**Affiliations:** Department of Orthopaedics, Sichuan Provincial People's Hospital, No. 32 West Second Section, First Ring Road, Chengdu 610072, China

## Abstract

Osteoporosis is one of the most common bone diseases, which is characterized by a systemic impairment of bone mass and fragility fractures. Age-related oxidative stress is highly associated with impaired osteoblastic dysfunctions and subsequent osteoporosis. In osteoblasts (bone formation cells), reactive oxygen species (ROS) are continuously generated and further cause lipid peroxidation, protein damage, and DNA lesions, leading to osteoblastic dysfunctions, dysdifferentiations, and apoptosis. Although much progress has been made, the mechanism responsible for oxidative stress induced cellular alternations and osteoblastic toxicity is still not fully elucidated. Here, we demonstrate that protein phosphatase 2A (PP2A), a major protein phosphatase in mammalian cells, mediates oxidative stress induced apoptosis in osteoblasts. Our results showed that lipid peroxidation products (4-HNE) may induce dramatic oxidative stress, inflammatory reactions, and apoptosis in osteoblasts. These oxidative stress responses may ectopically activate PP2A phosphatase activity, which may be mediated by inactivation of AKT/mTOR pathway. Moreover, inhibition of PP2A activity by okadaic acid might partly prevent osteoblastic apoptosis under oxidative conditions. These findings may reveal a novel mechanism to clarify the role of oxidative stress for osteoblastic apoptosis and provide new possibilities for the treatment of related bone diseases, such as osteoporosis.

## 1. Introduction

Bone remodeling is a highly dynamic physiological process that constantly responds to altered demand for structural support [1–3]. Osteoblasts (bone forming cells) and osteoclasts (bone resorbing cells) work simultaneously to maintain bone density and strength [4]. During aging, an impaired osteoblastic bone formation increased by decreased number and activity of individual osteoblastic cells and finally leads to osteoporosis [5]. Thus, osteoblast apoptosis increased by associating with inflammation-mediated osteoporosis, and oxidative stress might play an important role in these processes.

In osteoblasts, oxidative stresses may result in lipid peroxidation, protein damage, DNA lesions, and inflammatory responses, finally leading to apoptosis. Nowadays, it is widely accepted that aging increases oxidative stresses and osteoblast apoptosis [6, 7]. For example, oxidative stresses may induce osteoblast apoptosis by activating c-Jun N-terminal kinase (JNK) pathway, which causes cell injuries and reduces the number and function of osteoblasts, thereby inhibiting bone formation [8]. However, forkhead box O- (FoxO-) dependent oxidative defense might provide a mechanism to handle the oxygen free radicals constantly generated by the aerobic metabolism of osteoblasts and is thereby indispensable for bone mass homeostasis [9]. Moreover, oxidative stress may activate nuclear factor-*κ*B (NF-*κ*B) pathway to increase targeted gene transcriptions for inflammations [7]. Although these pathways or transcription factors have been identified in oxidative stress induced osteoblastic apoptosis, how oxidative stress regulates osteoblast survival needs further elucidation.

Protein phosphatase 2A (PP2A) is one of four major cytoplasmic serine/threonine phosphatases that is known to control diverse cellular process, including metabolism, kinase cascade, cell growth, and apoptosis [10, 11]. PP2A may play a positive regulatory role in activating proapoptotic and inhibiting antiapoptotic proteins by dephosphorylation. In the meantime, oxidative stress may induce PP2A activity to dephosphorylate pocket proteins pRb, p107, and p130 and promote apoptosis [12]. Many recent studies characterized specific PP2A substrates to identify its role in the apoptotic and other signal transduction pathways. For example, PP2A may dephosphorylate and inactivate antiapoptotic B-cell lymphoma-2 (BCL-2) protein [13]. Again, PP2A can activate proapoptotic Bad protein [14]. Moreover, PP2A has been reported to work as a substrate of caspase-3. Cleaving by caspase-3 results in an increase of PP2A activity [15]. All these studies identified PP2A as a proapoptotic factor. Thus, inhibition of PP2A activity by okadaic acid may prevent cell apoptosis [16, 17]. However, it has also been reported that okadaic acid induces apoptotic cell death in several types of cells [18, 19]. These conflicting studies raise a question that the molecular mechanism and effects of PP2A in cell apoptosis need to be further clarified.

In the present study, we propose to determine whether PP2A is involved in oxidative stress induced apoptosis in osteoblasts. Our results suggested that oxidative stress induced by 4-Hydroxynonenal (4-HNE) [20] increases dramatic osteoblastic apoptosis and inflammatory reactions. These oxidative stresses may activate PP2A phosphatase activity, which may be mediated by inactivation of AKT/mTOR pathway. Moreover, inhibition of PP2A activity by okadaic acid may partly prevent osteoblastic apoptosis under oxidative conditions. Our findings have established that PP2A might be a key regulator of oxidative stress induced apoptosis in osteoblasts. Our work will help to understand the regulatory mechanism of osteoblast homeostasis and offer a novel target for improving ROS-mediated osteoblast death and osteoporosis.

## 2. Materials and Methods

### 2.1. Cell Culture and Pharmacological Manipulations

Primary mouse osteoblasts were from individual calvaria of newborn wild-type pups and isolated according to reported assays [21, 22]. Briefly, calvaria was incubated in *α*-Minimum Essential Medium (*α*MEM, Gibco-Invitrogen, Carlsbad, CA) plus collagenase and trypsin (Sigma-Aldrich, St. Louis, MO, USA) at 37°C and cut into pieces for further digestion. Then cells were allowed to attach for 48 h and were then plated in precoated plates with 10% fetal bovine serum (FBS, Hyclone, Logan, Utah) and 1% antibiotics. Hereafter the medium was replaced every two days. Cultures were maintained and treated with gradient 4-HNE (Biomol, Hamburg, Germany) treatments (10 and 50 *μ*M for 0.5 to 2 h). Then these cells were harvested for subsequent experiments.

### 2.2. MTT and Hoechst Staining Assays

For MTT assay, 1 × 10^3^ cells were seeded in 96-well plates. On the next day, cells were incubated with 0 *μ*M (control), 10 *μ*M, and 50 *μ*M 4-HNE for 0.5, 1, and 2 h, respectively. Then viable cells were stained by adding 20 *μ*L MTT solution (5 mg/mL) per 100 *μ*L of growth medium. After incubating for 4 hours at 37°C, the media were removed and 150 *μ*L Dimethyl Sulfoxide (DMSO) was added to dissolve the formazan. The absorbance of each well was measured by microplate reader and viable cells are presented as a percent of the control. For Hoechst staining, 1 × 10^5^ osteoblasts were seeded in 6-well plates. Then cells were incubated with consistent 4-HNE and stained with Hoechst kit (Apoptosis-Hoechst staining kit, Beyotime Biotechnology, China). The cell counting was carried out using the software ImageJ from the National Institutes of Health.

### 2.3. Western Blots

The proteins were extracted from cells treated with 4-HNE by lysis buffer of 2% SDS, cocktail protease inhibitors, and phosphatase inhibitors (1.0 mM Na_3_VO_4_, 1.0 mM DTT, and 1.0 mM PMSF). The concentration of proteins was measured with BCA Protein Assay kit (Thermo Fisher Scientific Inc., Barrington, IL, USA). Then, these proteins were separated by SDS-PAGE and transferred onto PVDF (Bio-Rad) membranes following the standard procedures. Next, the membranes were blocked by 5% nonfat dry milk in PBST (PBS with 0.1% Tween 20) and incubated with anti-GAPDH, anti-pro-/cleaved-caspase-3, anti-BcL-2, anti-Bax (Billerica, MA, USA), anti-pAKT, anti-AKT, anti-pp70S6K, anti-p70S6K, anti-PP2A-a, anti-PP2A-b′, and PP2A-c-*α* (Cell Signaling Technology, Beverly, MA, USA) and appropriate secondary antibodies conjugated with horseradish peroxidase and developed with ECL Plus luminescent reagents (Thermo Fisher Scientific Inc., Barrington, IL, USA). The protein level quantification was also carried out by ImageJ.

### 2.4. Real-Time PCR Assay

Total RNAs were extracted from cells using RNA extraction kit (GeneAnswer, Zhengzhou Ansai Biotechnology Co., Zhengzhou, China). RNA was subjected to reverse transcription with reverse transcriptase as per manufacturer's instructions (Fermentas, USA). Quantitative real-time PCR was performed using the Bio-Rad iQ5 system using Bio-Rad proprietary iQ5 software (Hercules, CA, USA), and the relative gene expression was normalized to internal control as *β*-actin. Primer sequences for SYBR Green probes of target genes were as follows:* IL-1β*: CTGGTGTGTGACGTTCCCATTA and CCGACAGCACGAGGCTTT;* TNF-α*: CATCTTCTCAAAATTCGAGTGACA and TGG GAGTAGACAAGGTACAACCC; *β*-actin: GAGACCTTCAACACCCCAGC and ATGT CACGCACGATTTCCC.

### 2.5. PP2A Phosphatase Assay

The osteoblastic PP2A phosphatase activity was measured by Promega Serine/Threonine Phosphatase Assay System V2460 (Promega, Madison, WI) [23–25]. Briefly, primary osteoblasts were harvested to remove endogenous phosphate using provided spin columns. Then cell lysis was mixed with reaction premixes and enzymes to initiate these reactions. Next, equal volume of the molybdate dye/additive mixture was added to all wells to stop the reactions. After incubation for 15 min, PP2A activity was measured by absorbance at 600 nm with a plate reader. All measured values were normalized to control and we carried out statistical analysis.

### 2.6. Statistical Analysis

All data were expressed as mean ± standard deviation (*X* ± s) and analyzed by SPSS software (SPSS 16.0). The comparisons between groups were done using ANOVA tests for comparisons. The value of 0.05 (*∗*), 0.01 (*∗∗*), and 0.001 (*∗∗∗*) was assumed as the level of significance for the statistic tests.

## 3. Results

### 3.1. Oxidative Stress Induces Cell Apoptosis in Osteoblasts

**Figure 1:**
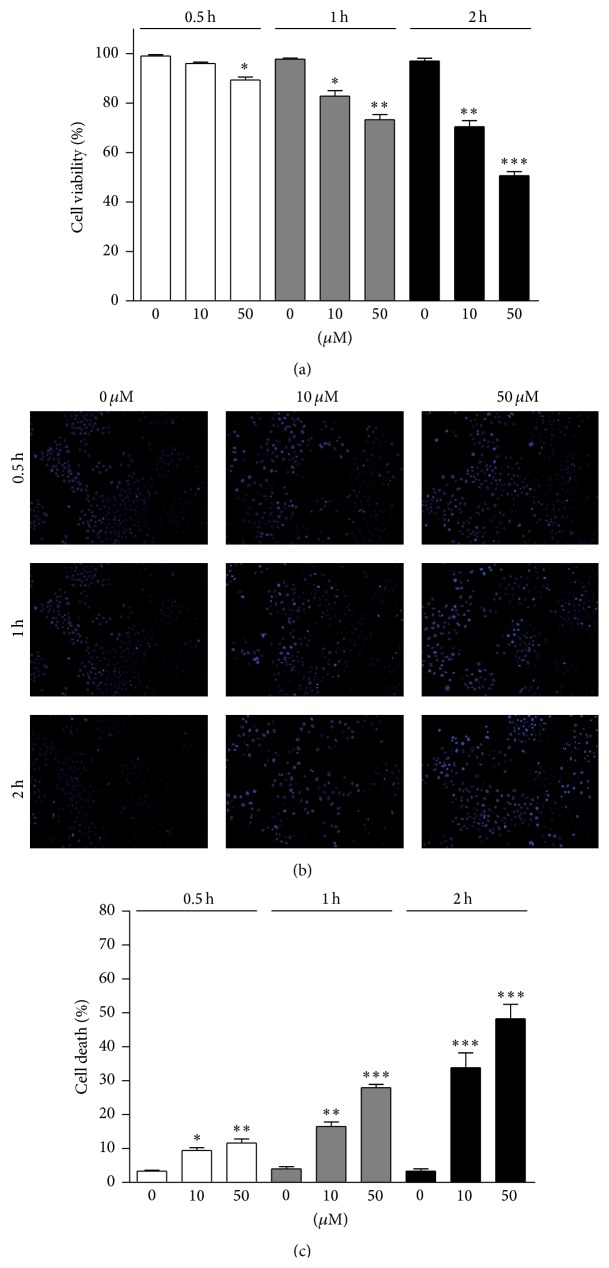
Oxidative stress induces cell apoptosis in osteoblasts. (a) MTT assay results indicate that osteoblastic viability (%) was downregulated by 4-HNE treatment (10 or 50 *μ*M for 0.5 to 2 h). ((b)-(c)) Hoechst staining and quantifications confirmed that 4-HNE treatment may induce dramatic osteoblastic apoptosis. Results are averages of three independent experiments. Data represent mean ± SEM. ^*∗*^ *P* < 0.05, ^*∗∗*^ *P* < 0.01, and ^*∗∗∗*^ *P* < 0.001.

To detect whether oxidative stress may induce cell apoptosis in primary osteoblasts, we detected the cell viability by 4-HNE treatment. Cell viability was determined by MTT assay, showing that increased 4-HNE concentrations (e.g., 50 *μ*M) and treatment times (e.g., 2 h) reduce cell viability in osteoblasts (Figure 1(a)). To confirm cell death in osteoblasts, we further examined osteoblastic apoptosis by Hoechst staining. Results showed that numbers of apoptotic cells were increased with the increasing of 4-HNE concentrations and extension times, especially in 50 *μ*M/2 h group (Figures 1(b) and 1(c)). Thus, these results indicate that lipid peroxidation mediated oxidative stress may effectively impair cell survival in primary osteoblasts.

### 3.2. Oxidative Stress Activates Apoptotic Pathways and Inflammatory Reactions in Osteoblasts

**Figure 2:**
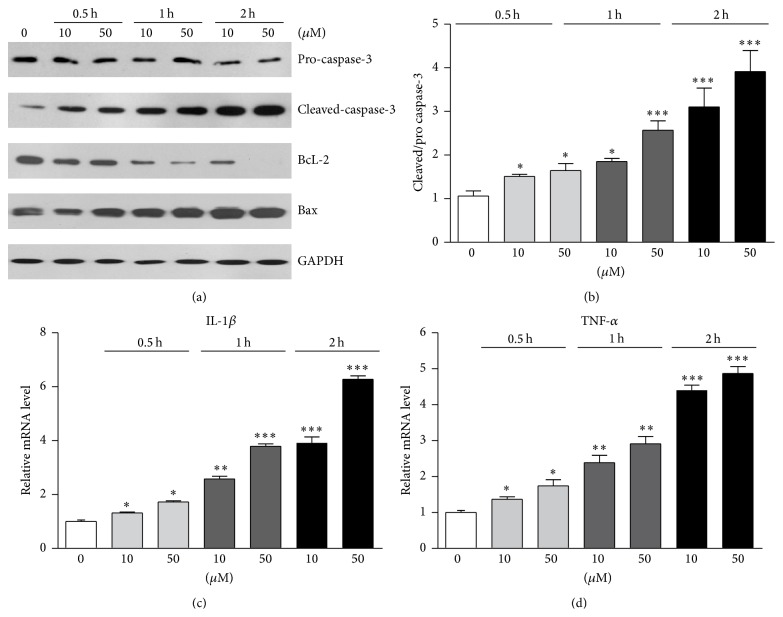
Oxidative stress activates apoptotic pathways and inflammatory reactions in osteoblasts. ((a)-(b)) Western blots results showed that apoptotic proteins cleaved-caspase-3 was induced by 4-HNE treatment. The proapoptotic protein Bax was also increased and antiapoptotic protein BcL-2 decreased. ((c)-(d)) Real-time PCR results suggest that transcription levels of* IL-1β* and* TNF-α*, osteoblastic inflammation genes, were upregulated by 4-HNE treatment. Results are averages of three independent experiments. Data represent mean ± SEM. ^*∗*^ *P* < 0.05, ^*∗∗*^ *P* < 0.01, and ^*∗∗∗*^ *P* < 0.001.

Caspase-3 is a key molecule to mediate apoptosis, and cleaved-caspase-3 is activated at early stages of apoptosis and eventually leads to apoptosis. To confirm the apoptotic effects of lipid peroxidation on osteoblasts, we examined protein levels of cleaved-caspase-3 in 4-HNE treated osteoblasts. Results showed that the levels of cleaved-caspase-3 were increased by 4-HNE treatment, and the expression level was extremely high by 50 *μ*M 4-HNE treatment for 2 h (Figures 2(a) and 2(b)). Moreover, BcL-2 is a well-known antiapoptotic protein while Bax is a proapoptotic protein. The protein level alterations of BcL-2/Bax affect cell survival. We found that the protein levels of BcL-2 were gradually decreased with the incubation with 4-HNE, while the protein levels of Bax increased (Figure 2(a)). Since oxidative stresses are highly correlated with inflammatory responses, we further investigated whether lipid peroxidation induced inflammation in osteoblasts. Real-time PCR results showed 4-HNE treatment could dramatically increase inflammatory gene transcriptions (e.g., IL-1*β* and TNF-*α*), which may contribute to the apoptosis in osteoblasts (Figures 2(c) and 2(d)). These findings confirm that lipid peroxidation could activate apoptotic pathways and inflammatory reactions in osteoblasts.

### 3.3. Protein Phosphatase 2A Is Activated by Oxidative Stress* via* AKT/mTOR Inactivation in Osteoblasts

**Figure 3:**
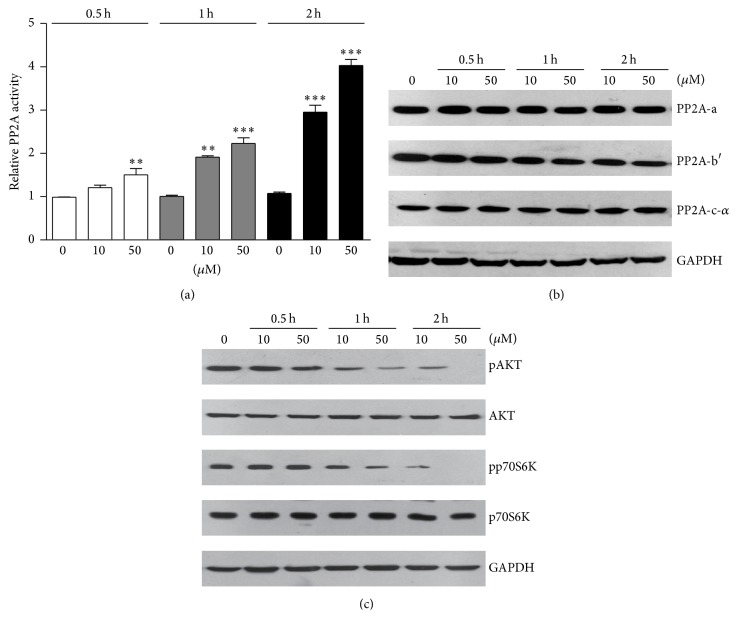
Protein phosphatase 2A is activated by oxidative stress* via* AKT/mTOR inactivation in osteoblasts. (a) Biochemical assays indicate that PP2A phosphatase activity was induced by 4-HNE treatment in osteoblasts. (b) Western blots showed that protein levels of PP2A three subunits were not altered by 4-HNE treatment, indicating that 4-HNE activates PP2A not by increasing its protein levels. (c) Western blots showed that AKT/mTOR pathways, indicated by pAKT and pp70S6K, are upregulated by 4-HNE treatment, which may cause PP2A activation. Results are averages of three independent experiments. Data represent mean ± SEM. ^*∗∗*^ *P* < 0.01 and ^*∗∗∗*^ *P* < 0.001.

To identify how lipid peroxidation mediated oxidative stress induces osteoblastic apoptosis, we focused on protein phosphatase 2A (PP2A) pathways, which was reported to control cell apoptosis and survival. For the first, we assayed the phosphatase activity of PP2A. Biochemical results showed that PP2A activity was dramatically increased by 4-HNE treatments, in a dose- and time-dependent manner (Figure 3(a)). PP2A consists of a dimeric core enzyme composed of the structural A and catalytic C subunits and a regulatory B subunit. Thus, we examined whether the components of PP2A complex were altered by lipid peroxidation products. Western blot results showed three subunits of PP2A, including PP2A-a, PP2A-b′, and PP2A-c, were not affected by 4-HNE treatment (Figure 3(b)). Thus, we propose that oxidative stress may induce PP2A activity* via* inactivation of its upstream inhibitors, such as AKT/mTOR pathway. Hence, we further examined whether AKT/mTOR pathway was inactivated under oxidative stress conditions. Results showed that protein levels of pAKT and pp70S6K (indicators of AKT/mTOR pathway) are decreased by 4-HNE treatment in osteoblasts (Figure 3(c)). Based on the above results, our findings suggested that lipid peroxidation products may activate PP2A phosphatase activity* via* AKT/mTOR inactivation in osteoblasts.

### 3.4. Inhibition of PP2A Activity May Partly Protect Osteoblasts from Apoptosis

**Figure 4:**
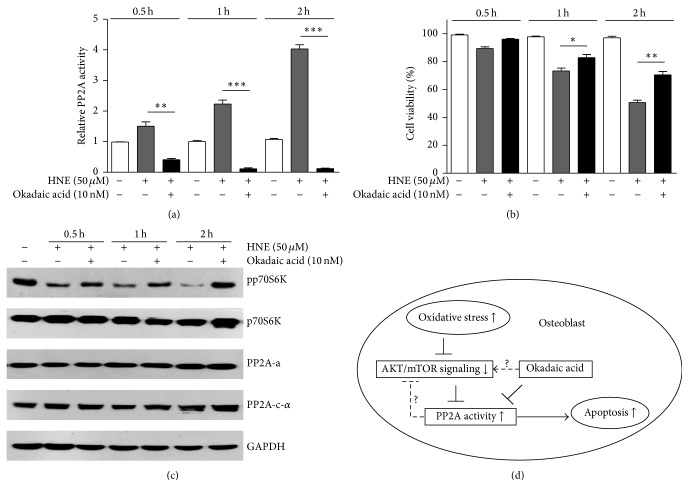
Inhibition of PP2A activity may partly protect osteoblasts from apoptosis. (a) PP2A activity assay showed that okadaic acid treatment could inhibit PP2A activity under oxidative conditions. (b) MTT assay showed that osteoblastic viability may be partly rescued by inhibition of PP2A activity* via* okadaic acid. (c) Western blots showed that okadaic acid may recover mTOR activity, indicated by pp70S6K, and not alter PP2A components protein levels. (d) Schematic representation explaining the network of oxidative stress, AKT/mTOR, PP2A, and cell apoptosis in osteoblasts. Oxidative stress may induce dramatic apoptosis in primary osteoblasts. The apoptotic signaling activation was closely associated with activation of PP2A activity. However, inhibition of PP2A activity may partly rescue osteoblastic apoptosis under oxidative stresses.

Earlier reports showed that 4-HNE treatment may induce PP2A activity in parallel with osteoblastic apoptosis, and we wonder whether inhibition of PP2A activity may restore the viability of osteoblasts after 4-HNE treatment. Here, we applied pretreatment of okadaic acid (inhibitor of PP2A) to 4-HNE treated osteoblasts. By PP2A phosphatase activity assay, we validated that okadaic acid indeed prevents PP2A activation under oxidative stress conditions (Figure 4(a)). Next, we assayed osteoblast viability* via *MTT assays. Results showed consistent increasing of cell survival with decreased PP2A activity in osteoblasts (Figure 4(b)). Further, we found that mTOR pathway activity, indicated by pp70S6K, was recovered after okadaic acid treatment, without alterations of PP2A subunits (Figure 4(c)). Collectively, these findings supported the notion that inhibition of PP2A activity may partly protect osteoblasts from oxidative stress mediated apoptosis.

## 4. Discussion

Oxidative stress has long been appreciated as an inducer of various bone related diseases, including osteoblastic death and osteoporosis. However, molecular mechanism of how oxidative stress mediates osteoblastic apoptosis is still not fully understood. In the present study, we reveal a novel mechanism to clarify that PP2A might be required for oxidative stress induced osteoblastic apoptosis. Our findings indicated that 4-HNE may induce dramatic inflammation and apoptosis in osteoblasts. Since PP2A is a key regulator of apoptosis in mammalian cells, we examined and found that PP2A activity may be significantly activated by 4-HNE treatment, which may be through inhibiting of AKT/mTOR pathway. Moreover, pharmacological inhibition of PP2A activity by okadaic acid may partly rescue the osteoblastic apoptosis accompanied with recovered mTOR pathway activity under oxidative stress conditions (Figure 4(d)). Our work may provide new possibilities for the treatment of osteoblastic diseases caused by oxidative stress.

Oxidative stress has been reported to induce cell apoptosis in both* in vitro *and* in vivo* experiments, targeted at both proapoptotic and antiapoptotic pathways. However, the relationship between oxidative stress and PP2A, a master phosphatase in mammalian cells, is not clearly clarified. It has been reported that oxidative stress may induce PP2A-dependent dephosphorylation of the pocket proteins, including pRb, p107, and p130 [12]. PP2A inhibitors, like okadaic acid, may block oxidative stress mediated ERK5 activation [26]. However, contradicting results showed that PP2A inhibitor okadaic acid was found to be a strong inducer of cellular H_2_O_2_ and superoxide production in distinct cell lines [27]. In the present study, our results showed that lipid peroxidation products induced oxidative stress may dramatically activate PP2A phosphatase activity (Figure 3), which contribute to the apoptosis in osteoblasts. Interestingly, it still needs further to study whether activated PP2A may turn over to enhance or inhibit oxidative stress.

When assaying the apoptotic effects of lipid peroxidation products on osteoblasts, we noticed a parallel increasing of apoptotic pathways and inflammation gene expressions (Figure 2). Considering inflammation is highly associated with cell viability [28, 29], we would like to investigate whether PP2A promotes apoptotic cascades* via *inflammation in the future study. Oxidative stress may activate apoptotic cascades, accompanied with inactivation of antiapoptotic pathways, such as AKT/mTOR [30]. Earlier reports showed that AKT/mTOR may act as an upstream regulator of PP2A. For example, insulin/mTOR pathway may result in a rapid inactivation of PP2A phosphatase activity [31]. Moreover, some results also suggested that mTOR regulate 4EBP1 and p70S6K phosphorylation* via* PP2A [32]. Thus, we identified that oxidative stress might gradually inactivate AKT/mTOR pathway, which may be responsible for activation of PP2A. Intriguingly, some reports showed that PP2A may turn over to regulate AKT and mTOR activity [33, 34]. In the present study, our results indicated that inhibition of PP2A activity by okadaic acid may recover osteoblasts survival accompanied with increased mTOR activity (Figure 4). These findings indicated that AKT/mTOR/PP2A may form a feedback loop to balance their activity. Nevertheless, the complicated network of PP2A and AKT/mTOR pathway need be further studied.

## 5. Conclusion

In summary, the present findings demonstrated that lipid peroxidation products mediated oxidative stress may induce dramatic apoptosis in primary osteoblasts. The apoptotic signaling activation was closely associated with ectopic activation of PP2A activity. However, inhibition of PP2A activity may partly rescue osteoblastic apoptosis under oxidative stress conditions. These findings will help to reveal that PP2A mediates oxidative stress induced apoptosis in osteoblasts, which provide a new thought for the clinical therapy of osteoblastic death related diseases, such as osteoporosis.
